# Can a central blood volume deficit be detected by systolic pressure variation during spontaneous breathing?

**DOI:** 10.1186/s12871-016-0224-z

**Published:** 2016-08-11

**Authors:** Michael Dahl, Chris Hayes, Bodil Steen Rasmussen, Anders Larsson, Niels H. Secher

**Affiliations:** 1 Department of Anesthesiology and Intensive Care Medicine, Aalborg University Hospital, Hobrovej 18-21, DK-9000 Aalborg, Denmark; 2 Hedenstierna laboratory, Section of Anesthesiology and Intensive Care, Department of Surgical Sciences, Uppsala University, ANIVA Ing. 70, 1. tv., S-75643 Uppsala, Sweden; 3 Department of Anesthesiology, The Copenhagen Muscle Research Center Rigshospitalet 2043, University of Copenhagen, Blegdamsvej 9, DK-2100 Copenhagen, Denmark

**Keywords:** Fluid responsiveness, Spontaneous breathing, Head-up tilt, Pulse pressure variation, Stroke volume variation, Systolic pressure variation

## Abstract

**Background:**

Whether during spontaneous breathing arterial pressure variations (APV) can detect a volume deficit is not established. We hypothesized that amplification of intra-thoracic pressure oscillations by breathing through resistors would enhance APV to allow identification of a reduced cardiac output (CO). This study tested that hypothesis in healthy volunteers exposed to central hypovolemia by head-up tilt.

**Methods:**

Thirteen healthy volunteers were exposed to central hypovolemia by 45° head-up tilt while breathing through a facemask with 7.5 cmH_2_O inspiratory and/or expiratory resistors. A brachial arterial catheter was used to measure blood pressure and thus systolic pressure variation (SPV), pulse pressure variation and stroke volume variation . Pulse contour analysis determined stroke volume (SV) and CO and we evaluated whether APV could detect a 10 % decrease in CO.

**Results:**

During head-up tilt SV decreased form 91 (±46) to 55 (±24) mL (mean ± SD) and CO from 5.8 (±2.9) to 4.0 (±1.8) L/min (*p* < 0.05), while heart rate increased (65 (±11) to 75 (±13) bpm; *P* < 0.05). Systolic pressure decreased from 127 (±14) to 121 (±13) mmHg during head-up tilt, while SPV tended to increase (from 21 (±15)% to 30 (±13)%). Yet during head-up tilt, a SPV ≥ 37 % predicted a decrease in CO ≥ 10 % with a sensitivity and specificity of 78 % and 100 %, respectively.

**Conclusion:**

In spontaneously breathing healthy volunteers combined inspiratory and expiratory resistors enhance SPV during head-up tilted induced central hypovolemia and allow identifying a 10 % reduction in CO. Applying inspiratory and expiratory resistors might detect a fluid deficit in spontaneously breathing patients.

**Trial registration:**

ClinicalTrials.gov number NCT02549482 Registered September 10^th^ 2015.

## Key messages

In spontaneous breathing healthy volunteers combined inspiratory and expiratory resistors enhance systolic pressure variation and allow for identifying a central volume deficit with a sensitivity and specificity of 78 % and 100 %, respectively. Combined inspiratory and expiratory resistors might help detecting a fluid deficit in spontaneously breathing patients.

## Background

Fluid therapy is an integrated part of emergency and critical care medicine as in anesthesia. However, there are few measurements that asses hypovolemia and consequently to what extent a patient is in need of fluid, i.e. responds with improved cardiovascular function after volume administration (being “fluid responsive”) [1]. Unfortunately, clinical judgment or, e.g. recording of central venous pressure [2–7] does not provide adequate information whether a patient is in need of intravascular volume expansion. In mechanically ventilated patients without cardiac arrhythmias exposed to a tidal volume larger than 8 mL/kg lean body weight, arterial pressure variation (APV) predicts volume responsiveness defined as an increase in stroke volume (SV) or cardiac output (CO) when the patient is exposed to an intravascular volume load [8–14]. In spontaneously breathing patients however, APV is insufficient to guide volume therapy [15–17] and thus volume therapy is guided by recording of SV and/or CO response or change in end-tidal CO_2_ tension , e.g. when the patient is exposed to passive raising the legs [16, 18–20] or Trendelenburg’s position [21]. Noteworthy, Zaniboni et al. [22] found a correlation for APV between mechanically ventilated patients and patients ventilated by spontaneous flow triggered synchronized intermittent mechanical ventilation.

Yet, APV can detect fluid responsiveness as demonstrated in swine breathing through an inspiratory and expiratory resistor that augment pulse pressure variations (PPV) [23] and in healthy volunteers with paced breathing and/or respiratory resistors [24]. Similarly, we considered whether the intra-thoracic pressure oscillations when amplified by inspiratory (increasing the negative intra-thoracic pressure) and expiratory resistors (increasing the expiratory intra-thoracic pressure) would allow detection of an intravascular volume deficit in humans. In this study, we tested that hypothesis in healthy humans exposed to a reduction in the central blood volume by head-up tilt. Separate evaluation was made by providing the subjects to an inspiratory resistance, to an expiratory resistance, or to both with no application of resistors serving as control. We aimed to identify which expression of APV is most sensitive to a significant reduction of the central blood volume resulting in a 10 % reduction in CO.

## Methods

Thirteen healthy volunteers (four women) 25 years (range 18–36) of age (Table 1) were recruited through www.forsogsperson.dk. Exclusion criteria were pregnancy, breast-feeding or use of any medication. The protocol was approved by the ethics committee for human research for The Capital region of Denmark (H-4-2010-110) in accordance with the Helsinki II declaration and oral and written informed consent was obtained.

**Table 1:** Characteristics of the subjects (*n* = 13)

Gender (F/M)	4/9
Age (years)	25 ± 5
Height (cm)	178 ± 10
Weight (kg)	73 ± 13
BMI (kg/m^2^)	23.0 ± 3.2
BSA (m^2^)	1.9 ± 0.2

Values are mean ± standard deviation

*BMI* body mass index, *BSA* body surface area

The volunteers were placed supine on a tilt table with heart rate monitored by a three-lead ECG and arterial oxygen saturation by pulse oximetry (SpO_2_) (Philips SpO2 Sensor M1191BL ViCare Medical, Denmark) on the right third finger of the dominant hand. A peripheral venous access was established and a 20 G arterial catheter was placed in the brachial artery of the non-dominant arm and both were maintained by infusion of isotonic saline (3 mL/h). The arterial catheter was connected to a transducer kept at heart level for registration of arterial pressure and stroke volume variation (SVV) (Vigileo-Flotrac™, version 1.07, Edwards Lifesciences, Nyon, Switzerland) as well as blood gas variables (ABL, Radiometer, Copenhagen). CO and the arterial pressure curve were stored for subsequent determination of arterial pulse pressure (PPV) and systolic pressure variation (SPV). Finally, a catheter was placed via a brachial vein and advanced to the subclavian vein to register central venous oxygen saturation (ScvO_2_) (ABL, Radiometer, Copenhagen).

**Fig. 1:**
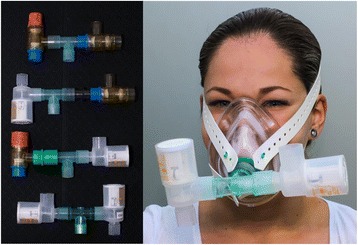
Left: four respiratory resistors: no resistance, expiratory resistance, inspiratory resistance, and both inspiratory and expiratory resistances. Right: facemask applied with combined inspiratory and expiratory resistors (Model photo)

The subjects were breathing spontaneously with respiratory rate determined by capnography (Philips CO_2_ Filterline, ViCare Medical, Denmark) and provided with a facemask (Intersurgical Ltd., Wokingham, Berkshire, UK) (Fig. 1) randomly fitted with an inspiratory resistor, an expiratory resistor, a combination of the two resistors, or with no resistors. Each resistor provided a 7.5 cmH_2_O threshold resistance (CPAP; Philips Respironics, Herrshing, Germany) and were applied for two minutes [18] with variables obtained in the second minute.

**Fig. 2:**
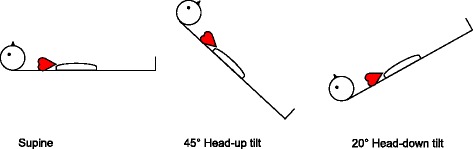
Three postures representing normovolemia (supine), central hypovolemia (head-up tilt), and central hypervolemia (head-down tilt)

Initially, variables were recorded with each resistor configuration while the subjects were supine (normovolemia). Secondly, the table was tilted 45° head-up to reduce the central blood volume hence simulating hypovolemia [25] (Fig. 2). Finally, 20° head-down tilt was used to expand the central blood volume and hence simulating mild hypervolemia [25, 26] . After each change of the tilt table position we allowed a 10 min equilibration time before variables were obtained.

PPV was ((PP_max_ – PP_min_)/((PP_max_ + PP_min_)/2)) × 100, where PP_max_ and PP_min_ are the maximal and minimal difference between systolic and diastolic pressure during the respiratory cycle, respectively [12] and SPV was calculated by an analogous formula. PPV and SPV were calculated from the stored recordings, while other variables were noted on-line.

### Statistics

For a 1-beta (power) of 0.8 and an alpha (*P*) of 0.05 and assuming an increase in arterial pressure variations by 10 % with a SD of 5 % by the intervention, a minimum of 8 subjects were needed. Statistics was performed with Stata 13.0 (StataCorp LP, Texas, USA) and QQ-plots identified that the data were normally distributed. Hemodynamic and respiratory responses were analyzed by using a two-way ANOVA with interaction between position and resistor. Estimation of fluid responsiveness was carried out using an ANOVA model with resistor as factor, only for head-up tilt, and Receiver Operating Characteristic (ROC) (Hanley and McNeil’s method). A *P* value < 0.05 was considered statistically significant.

## Results

### Hemodynamic responses and blood gas variables

From the supine position to head-up tilt CO decreased from 5.8 (±2.9) to 4.0 (±1.8) L/min (mean ± SD), SV from 91 (±46) to 55 (±24) ml, systolic pressure from 127 (±14) to 121 (±13) mmHg and ScvO_2_ from 0.79 (±0.07)% to 0.68 (±0.13)%, while diastolic pressure (64 (±7) to 69 (±6) mmHg) and heart rate (65 (±11) to 75 (±13) bpm) increased (*P* < 0.05). Similarly, from the supine position to head-down tilt there was a decrease in CO, SV and systolic pressure but no changes in heart rate, diastolic pressure or ScvO_2_ (Table 2). There were no changes in respiratory rate or SpO_2_ between the three body positions and only small changes in arterial blood gas variables and no significant interactions between position and respiratory resistor application.

**Table 2:** Hemodynamic and respiratory variables at three postures whatever respiratory resistor(s) applied

	Supine position	Head-up tilt	Head-down tilt
Cardiac output (L/min)	5.8 ± 2.9	4.0 ± 1.8*	5.1 ± 2.2*
Stroke Volume (mL)	91 ± 46	55 ± 24*	81 ± 36*
Systolic blood pressure (mmHg)	127 ± 14	121 ± 13*	120 ± 11*
Diastolic blood pressure (mmHg)	64 ± 7	69 ± 6*	65 ± 6
Heart rate (min^−1^)	65 ± 11	75 ± 13*	65 ± 11
Respiratory rate (min^−1^)	10 ± 4	10 ± 4	10 ± 3
Central venous oxygen saturation	0.79 ± 0.07	0.68 ± 0.13	0.79 ± 0.09
Ph	7.43 ± 0.03	7.45 ± 0.04*	7.44 ± 0.04
Oxygen partial pressure (kPa)	14.1 ± 1.6	14.3 ± 1.0	14.7 ± 1.6*
Carbondioxid partial pressure (kPa)	5.0 ± 0.6	4.6 ± 0.7*	4.8 ± 0.7*

Values are mean ± standard deviation. *P < 0.05 compared to the supine position

There was no interaction between position and application of resistors

### Detecting central hypovolemia

**Fig. 3:**
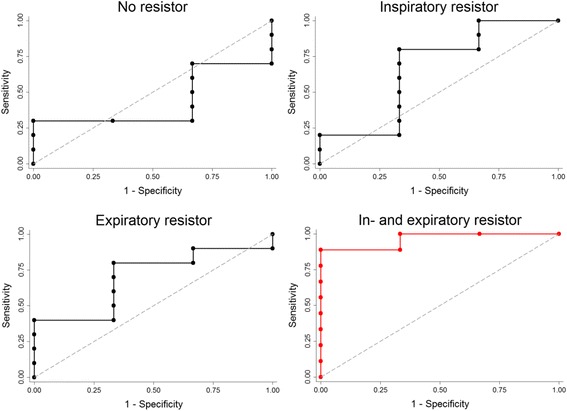
Receiver operating characteristic (ROC) curves during head-up tilt for systolic pressure variation (SPV) with the four different respiratory resistors. Area under the ROC curve 0.43 (0.10; 0.77 ) (confidence interval) for no resistor, 0.67 (0.25 ;1.00) for the inspiratory resistor, 0.70 (0.37; 1.00) for the expiratory resistor, and 0.96 (0.86; 1.00) for the combined inspiratory and expiratory resistor

**Fig. 4:**
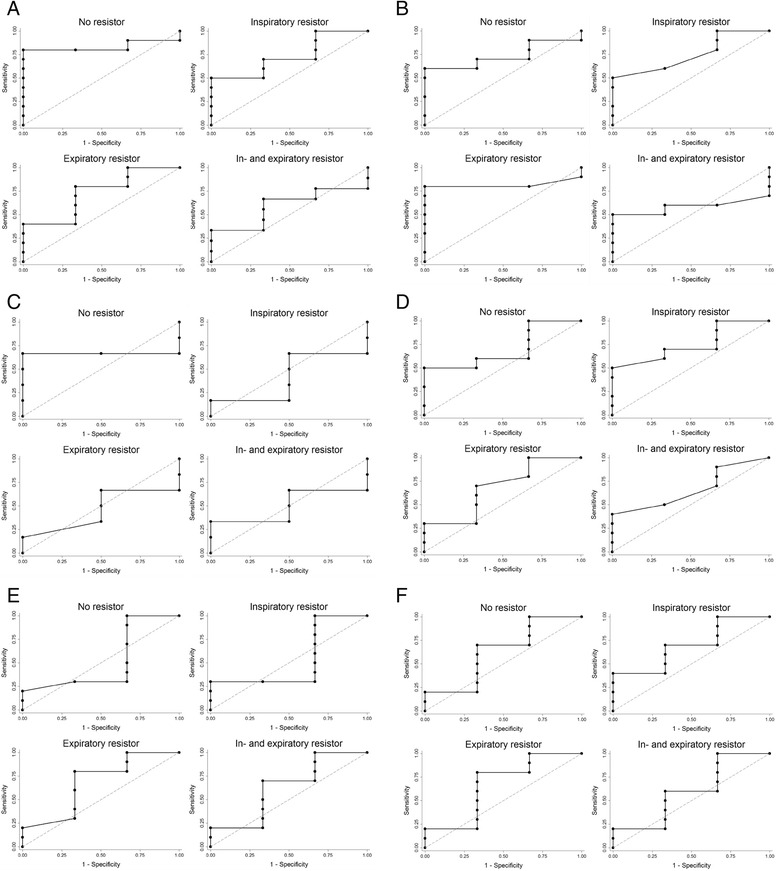
Receiver operating characteristic (ROC) curves during head-up tilt with the four different respiratory resistors. **a**: Pulse pressure variation (PPV) with area under the ROC curve 0.83 (0.61;1.00) (confidence interval) for no resistor, 0.73 (0.42;1.00) for the inspiratory resistor, 0.73 (0.40;1.00) for the expiratory resistor, and 0.59 (0.25;0.93) for the combined inspiratory and expiratory resistor. **b**: Stroke volume variation (SVV) with area under the ROC curve 0.73 (0.46;1.00) for no resistor, 0.75 (0.46;1.00) for the inspiratory resistor, 0.82 (0.58;1.00) for the expiratory resistor, and 0.58 (0.28;0.88) for the combined inspiratory and expiratory resistor. **c**: Central venous oxygen saturation (ScvO_2_) with area under the ROC curve 0.67 (0.29;1.00) for no resistor, 0.42 (0.00;0.88) for the inspiratory resistor, 0.46 (0.02;0.90) for the expiratory resistor, and 0.50 (0.08;0.92) for the combined inspiratory and expiratory resistor. **d**: Systolic blood pressure with area under the ROC curve 0.70 (0.37;1.00) for no resistor, 0.75 (0.46;1.00) for the inspiratory resistor, 0.68 (0.31;1.00) for the expiratory resistor, and 0.67 (0.34;0.99) for the combined inspiratory and expiratory resistor. **e**: Heart rate (HR) with area under the ROC curve 0.52 (0.08;0.95) for no resistor, 0.53 (0.11;0.96) for the inspiratory resistor, 0.68 (0.29;1.00) for the expiratory resistor, and 0.63 (0.22;1.00) for the combined inspiratory and expiratory resistor. **f**: Stroke volume (SV) with area under the ROC curve 0.63 (0.22;1.00) for no resistor, 0.70 (0.36;1.00) for the inspiratory resistor, 0.67 (0.25;1.00) for the expiratory resistor, and 0.60 (0.19;1.00) for the combined inspiratory and expiratory resistor

Ten volunteers showed a ≥ 10 % decrease in CO between the supine and head-up tilt positions. Regardless of tilt table position the combined inspiratory and expiratory resistors increased SVV, SPV and PPV, while the inspiratory resistor increased SPV and PPV and the expiratory resistor only SPV (Table 3). Sensitivity, specificity, positive predictive value, negative predictive value, area under the curve (AUC), and optimal cut-off for these variables, as well as ScvO_2_ are shown in Table 4. The best prediction of a central volume deficit (a 10 % reduction in CO) was obtained with SPV when the combined resistors were applied. For that configuration SPV tended to increase (from 21 (±15)% to 30 (±13)%) and revealed a sensitivity of 78 % and a specificity of 100 % with a positive predictive value of 100 %, a negative predictive value of 60 %, and an AUC of 0.96 (0.86;1.00) (confidence interval) (Fig. 3) when SPV was larger than 37 %. Figure 4, Panels a-f show ROC-curves for PPV, SVV, ScvO_2_, systolic blood pressure, heart rate and SV for comparison.

**Table 3:** Arterial pressure variations with different airway resistors during head-up tilt

	No resistor (%)	Inspiratory resistor (%)	Expiratory resistor (%)	Inspiratory/expiratory resistor (%)
Systolic pressure variation	17 ± 11	26 ± 14*	26 ± 18*	28 ± 14*
Stroke volume variation	15 ± 8	19 ± 8	23 ± 7*	29 ± 12*
Pulse pressure variation	7 ± 4	9 ± 6	8 ± 6	10 ± 6*

Values are mean ± standard deviation. *P < 0.05 compared to no resistor

**Table 4:** Sensitivity, specificity, positive predictive value and negative predictive value using 10 % difference in cardiac output between supine position to head-up tilt to define central hypovolemia

		AUC	Optimal cut-off (%)	Sensitivity (%)	Specificity (%)	Positive predictive value (%)	Negative predictive value (%)
Stroke volume variation							
	No resistor	0.73 (0.46;1.00)	13	60	100	100	43
	Expiratory resistor	0.82 (0.58;1.00)	28	70	100	100	50
	Inspiratory resistor	0.75 (0.46;1.00)	18	50	100	100	38
	Ins-/expiratory resistor	0.58 (0.28;0.88)	31	40	100	100	33
Systolic pressure variation							
	No resistor	0.43 (0.10;0.77)	19	20	100	100	27
	Expiratory resistor	0.70 (0.37;1.00)	33	70	67	88	40
	Inspiratory resistor	0.67 (0.25;1.00)	36	80	67	89	50
	Ins-/expiratory resistor	0.96 (0.86;1.00)	37	78	100	100	60
Pulse pressure variation							
	No resistor	0.83 (0.61;1.00)	7	80	100	100	60
	Expiratory resistor	0.73 (0.40;1.00)	4	70	67	88	40
	Inspiratory resistor	0.73 (0.42;1.00)	7	50	100	100	38
	Ins-expiratory resistor	0.59 (0.25;0.93)	12	67	67	86	40
Central venous oxygen saturation							
	No resistor	0.67 (0.29;1.00)	71	50	100	100	40
	Expiratory resistor	0.46 (0.02;0.90)	70	50	50	75	25
	Inspiratory resistor	0.42 (0.00;0.88)	77	50	50	75	25
	Ins-expiratory resistor	0.50 (0.08;0.92)	77	33	100	100	33

*AUC* area under the curve with confidence interval

## Discussion

In spontaneously breathing healthy volunteers application of a 7.5 cmH_2_O threshold resistance on both the inspiratory and expiratory side of a facemask during head-up tilt induced central hypovolemia enhanced the variation in arterial pressure during the respiratory cycle sufficiently to detect a 10 % reduction in CO. The highest sensitivity (78 %) and specificity (100 %) was observed for SPV with a threshold of 37 %. As a proof of principle, the results are in line with results by Bronzwaer et al. [24]. However, in contrast to the present findings that group found PPV to be superior to SPV. This difference may be due to a lower breathing rate in the Bronzwaer-study and hence larger tidal volume as well as blood pressure measurement by the non-invasive volume clamp method. Furthermore, we did not find any of the more commonly used variables, e.g. ScvO_2_, SV, heart rate or systolic blood pressure to be superior to SPV when the combined inspiratory and expiratory resistor was applied (Fig. 4, Panel a-f).

Head-up tilt [25, 27] as, e.g. lower body negative pressure, eventually combined with heat stress [28] reduces the central blood volume and has the advantage compared to a blood loss that the intervention can be terminated immediately if the subject becomes ill. That central hypovolemia was provoked by head-up tilt was indicated by a decrease in ScvO_2_ and an increase in heart rate [29]. We found CO and SV also to decrease during head-down tilt, however the reduction was so small that it did not affect ScvO_2_ significantly and neither Harms et al. [29] nor Bundgaard-Nielsen et al. [30] found a decrease in CO during head-down tilt and only a decrease in SV when the subjects were tilted 90° head-down. Similarly, moderate head-down tilt did not affect heart rate significantly [29, 30]. Variables were obtained after a ten-minute equilibration period in each body position with randomized application of the resistors. A shorter equilibration period, e.g. one minute, is probably enough to register pulse changes during tilt tests [27], but we decided to use a longer period to be sure that the central blood volume was displaced.

Our study has several limitations: First, we studied healthy volunteers who may not be representative for a hospitalized population. For example, in an ICU population only 50 % of patients increase CO ≥ 10 % when challenged with a fluid bolus [31]. Furthermore, the subjects were not fasting or told to abstain from heavy physical exercise and caffeinated beverages prior to the experiment. Secondly, our test was “the reverse” of the clinical practice; i.e. we provoked central hypovolemia by tilting the subjects head-up and evaluated the change in CO and arterial pressure variations, and did not study whether these changes would be corrected by fluid administration. The CO decreased by more than 10 % in 10 of 13 subjects when exposed to 45° head-up tilt and a larger tilt angle would likely result in a more significant reduction of CO. However, we used a relatively long equilibration period. Thirdly, we used an uncalibrated pulse contour technic to detect SV and CO [32]. Fourthly, the results depend not only on the resistance of the resistors, but also on the respiratory effort by the subjects. The threshold resistance was set at 7.5 cmH_2_O and chosen because that level is in accordance with an animal study using SPV to indicate hypovolemia [23]. An airway threshold resistor between 5 and 10 cmH_2_O is used for positive end-expiratory pressure or continuous positive airway pressure and is accepted by most patients. Finally, we did not control the breathing rate. A fixed slow paced breathing might have enhanced the results as demonstrated by Zöllei et al. [33] and Bronzwaer et al. [24].

## Conclusion

Applying inspiratory and expiratory resistors to spontaneously breathing healthy volunteers allows for identifying significant central hypovolemia by recording of systolic pressure variations.

The clinical implication of the results is that systolic pressure variations might be used to detect a volume deficit in spontaneously breathing patients.

## Abbreviations

APV, arterial pressure variation; CO, cardiac output; CVP, central venous pressure; PP_max_, maximal pulse pressure; PP_min_, minimal pulse pressure; PPV, pulse pressure variation; ScvO_2_, central venous oxygen saturation; SPV, systolic pressure variation; SV, stroke volume; SVV, stroke volume variation
